# Hospital Sunday Fund

**Published:** 1909-01-23

**Authors:** 


### HOSPITAL SUNDAY FUND.

THE collections made in the churches and chapels of the metropolis for the London Hospital Sunday Fund last year were £2,547 less than the sum raised in 1907. Among the principal amounts included in the total of £40,239 16s. are :

| | £ | s. | d. |
|---|---|---|---|
| Church of England ... ... ... | 31,914 | 1 | 3 |
| Congregationalists ... ... ... | 1,644 | 2 | 10 |
| Jews ... ... ... ... ... ... | 1,491 | 11 | 6 |
| Presbyterians ... ... ... | 1,097 | 17 | 1 |
| Wesleyans ... ... ... ... | 916 | 10 | 3 |
| Baptists ... ... ... ... | 739 | 7 | 2 |
| Roman Catholics ... ... ... | 646 | 4 | 10 |
| Unitarians ... ... ... ... | 385 | 0 | 9 |
| Church of Scotland ... ... ... | 308 | 7 | 10 |
| Theistic Church ... ... ... | 210 | 0 | 0 |

St. Paul's Cathedral again headed the list with a collection of £4,510, while St. Michael's, Chester Square, sent £1,187, and Christ Church, Lancaster Gate, £1,003.

